# Interleukin-1 Blockers: A Paradigm Shift in the Treatment of Recurrent Pericarditis

**DOI:** 10.3390/life14030305

**Published:** 2024-02-26

**Authors:** Emilia Lazarou, Christos Koutsianas, Panagiotis Theofilis, George Lazaros, Dimitrios Vassilopoulos, Charalambos Vlachopoulos, Costas Tsioufis, Massimo Imazio, Antonio Brucato, Dimitris Tousoulis

**Affiliations:** 1 First Cardiology Department, School of Medicine, Hippokration General Hospital, National and Kapodistrian University of Athens, Vas. Sofias 114, 11527 Athens, Greece; emilazarou@gmail.com (E.L.); panos.theofilis@hotmail.com (P.T.); glaz35@hotmail.com (G.L.); cvlachop@otenet.gr (C.V.); ktsioufis@gmail.com (C.T.); 2 Clinical Immunology-Rheumatology Unit, 2nd Department of Medicine and Laboratory, Joint Academic Rheumatology Program, School of Medicine, Hippokration General Hospital, National and Kapodistrian University of Athens, 114 Vass. Sophias Ave., 11527 Athens, Greece; ckoutsianas@gmail.com (C.K.); dvassilop@med.uoa.gr (D.V.); 3 Cardiothoracic Department, Santa Maria della Misericordia University Hospital, 33100 Udine, Italy; massimo_imazio@yahoo.it; 4 Department of Biomedical and Clinical Sciences, University of Milan, 20157 Milan, Italy; antonio.brucato@unimi.it

**Keywords:** interleukin-1 blockers, recurrent pericarditis, steroid dependence, pathophysiology, NLRP3 inflammasome

## Abstract

Recurrent pericarditis is a problematic clinical condition that impairs the quality of life of the affected patients due to the need for repeated hospital admissions, emergency department visits, and complications from medications, especially glucocorticoids. Unfortunately, available treatments for recurrent pericarditis are very limited, including only a handful of medications such as aspirin/NSAIDs, glucocorticoids, colchicine, and immunosuppressants (such as interleukin-1 (IL-1) blockers, azathioprine, and intravenous human immunoglobulins). Until recently, the clinical experience with the latter class of medications was very limited. Nevertheless, in the last decade, experience with IL-1 blockers has consistently grown, and valid clinical data have emerged from randomized clinical trials. Accordingly, IL-1 blockers are a typical paradigm shift in the treatment of refractory recurrent pericarditis with a clearly positive cost/benefit ratio for those unfortunate patients with multiple recurrences. A drawback related to the above-mentioned medications is the absence of universally accepted and established treatment protocols regarding the full dose administration period and the need for a tapering protocol for individual medications. Another concern is the need for long-standing treatments, which should be discussed with the patients. The above-mentioned unmet needs are expected to be addressed in the near future, such as further insights into pathophysiology and an individualized approach to affected patients.

## 1. Introduction

The most common pericardial syndromes in clinical practice include acute pericarditis (either first episode or recurrences), constrictive pericarditis, and isolated pericardial effusion without evidence of pericardial inflammation (namely pericarditis) [1,2,3,4,5,6,7,8,9,10]. Pericardial syndromes have recently, in the context of the coronavirus disease (COVID-19) era, come to the attention of both clinicians and media because inflammatory heart disease (myocarditis and/or pericarditis) may complicate SARS-CoV-2 infection and vaccination against COVID-19, especially with the mRNA platforms [11,12,13,14,15,16].

Recurrent pericarditis develops in 15–30% of cases after an episode of acute pericarditis [5,17,18]. Unfortunately, not uncommonly after the first episode, a vicious circle starts with further pericarditis recurrences developing afterward [1,19,20].

In particular, the rate of a second recurrence is estimated to be 25–50% (with lower rates observed in patients receiving colchicine). A third recurrence is observed in 20–40% of cases. Notably, approximately 6% of patients with recurrent disease will develop multiple recurrences (≥3) with a median duration of disease activity estimated between 2.8 and 4.7 years [6,21,22,23,24]. This troublesome subset of patients sometimes depicts an incessant clinical course and falls into the so-called colchicine-resistant glucocorticoid-dependent recurrent pericarditis group [5]. These unfortunate patients develop recurrences every time they attempt to lower the dose of cortisone below a threshold, which is rather specific for the individual patient [25,26,27].

The above-mentioned patient category is exposed to the side effects of chronic steroid use, which is proportional to the dose required to achieve pericarditis remission [28]. The problem is even more pronounced in children with recurrent pericarditis needing chronic steroid treatment for refractory pericarditis due to the negative impact of steroid treatment on growth [29]. Alternative treatments proposed for this disorder were steroid-sparing agents; however, strong data on their overall efficacy are lacking [1,30,31,32]. Fortunately, the introduction of interleukin-1 (IL-1) blockers in the present decade constituted a paradigm shift towards the targeted therapy of colchicine-resistant glucocorticoid-dependent recurrent pericarditis [33,34,35,36,37,38,39,40,41,42,43].

In this narrative review, we will briefly discuss the pathogenesis of this problematic entity and treatment options, emphasizing the IL-1 blocker mechanism of action, treatment protocols, safety, and efficacy, along with some controversial issues in certain clinical circumstances.

## 2. Pathophysiology of Recurrent Pericarditis

For many years, the underlying mechanism of recurrent pericarditis included infections (reactivation of the initial infection or reinfection by a new agent) and autoimmunity, with the two mechanisms not necessarily mutually exclusive but sometimes complementary [1,5,44,45,46,47]. Remarkably, in recent years, a third mechanism has been added as a contributor to the pathogenesis of recurrent pericarditis to the already proposed, namely the autoinflammatory mechanism [48,49,50,51]. Autoinflammatory diseases constitute a heterogeneous category of diseases that are characterized by periodic inflammation mediated by the inflammasome [9]. Clinical manifestations include repetitive attacks of sterile inflammation of several tissues, including joints, skin, and serosal surfaces, due to an abnormal innate immune system activation as a result of a variety of both exogenous and endogenous stimuli [5].

Typical examples of diseases attributed to autoinflammatory mechanisms encompass familial Mediterranean fever (FMF), tumor necrosis factor receptor-associated periodic syndrome (TRAPS), and cryopyrin-associated periodic syndromes (CAPS) [1,5,9,52,53]. The common denominator of the latter disorders is an exaggerated production of IL-1 due to a dysregulated activation of the NLRP3 inflammasome [1,5,9,50,54,55,56,57].

Interestingly, we may assume the underlying mechanism based on the clinical presentation (the so-called clinical phenotype). Serositis (pleuro-pericarditis) and fever are compatible with an underlying autoinflammatory phenotype in specific cases presenting with prominent CRP elevation. In contrast, patients presenting during the acute attack of pericarditis with low-moderate peak CRP levels along with the presence of a combination of arthritis, sicca syndrome, Raynaud phenomenon, and elevated serum autoantibodies are most probably affected by an autoimmune disorder [5,9,49]. The recognition of the underlying mechanism has important clinical implications since patients depicting the autoinflammatory phenotype have an impressive response to IL-1 blockers [5,49].

As already mentioned, in recent years, recurrent pericarditis has been regarded in several instances as an IL-1-mediated disease. Two subtypes of IL-1, namely IL-1α and IL-1β, bind to the type 1 IL-1 receptor and control hematopoiesis, inflammation, and immune responses [58]. Since IL-1α is primarily membrane-bound, its inflammatory effects are localized in the pericardium, causing inflammation and occasionally fibrosis, which occasionally may progress to constrictive pericarditis [1,5,9,58]. Conversely, the predominant circulating form of IL-1 is IL-1β, which accounts for IL-1 systemic effects such as fever, serositis, elevation of acute phase reactants, etc. [58]. The NLRP3 inflammasome, through its main product, IL-1β, plays a central role in clinical manifestations [1,5,9,50,54,55,56,57,58].

**Figure 1:**
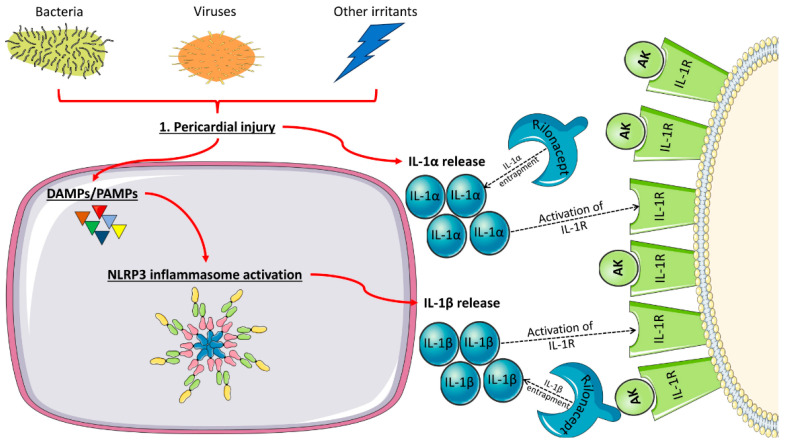
The role of interleukin-1 and NLRP3 inflammasome in pericarditis and the mechanism of action of IL-1-targeted therapeutics. DAMP: damage-associated molecular pattern; PAMP: pathogen-associated molecular pattern; IL: interleukin; IL-1R: interleukin-1 receptor; and AK: anakinra.

While the role of the IL-1 and NLRP3 inflammasome has been well-studied in the setting of atherosclerosis, their impact on pericardial diseases has been investigated to a lesser extent [59,60]. Therefore, we can assume that pericardial injury leads to the release of IL-1α and damage or pathogen-associated molecular patterns, which in turn activate the NLRP3 inflammasome (Figure 1). Its activation promotes the release of IL-1β. IL-1 subtypes then activate the IL-1 receptor, resulting in an enhanced inflammatory response [1,5,9,50,54,55,56,57,58,61,62].

In favor of the above-mentioned series of events in the zymosan-induced mouse model of acute pericarditis, an increased expression in the NLRP3 inflammasome was detected along with the classical features of pericardial inflammation, namely pericardial effusion and pericardial thickening due to edema. On the other hand, neutralization of IL-1α and 1β with an IL-1 trap reduced NLRP3 inflammasome expression and pericardial inflammation in the same experimental model [63].

## 3. Treatment of Recurrent Pericarditis

**Figure 2:**
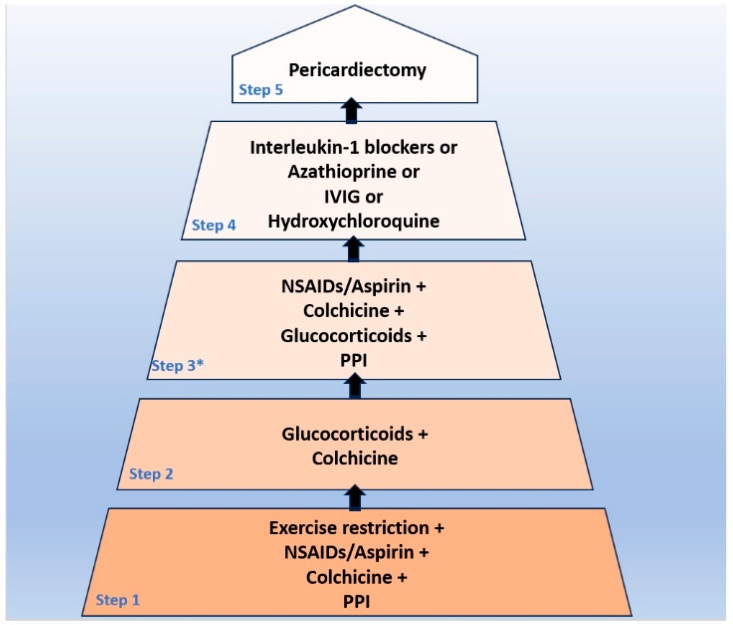
Proposed algorithm for the treatment of patients with recurrent pericarditis according to the 2015 European Society of Cardiology Guidelines for the diagnosis and management of pericardial diseases. PPI = protein pump inhibitors; NSAIDs = non-steroidal anti-inflammatory drugs; and IVIG = intravenous immunoglobulin. * This step consists of administering intermediate doses of aspirin/NSAIDs—steroids (and a standard dose of colchicine) to achieve remission, avoiding side effects related to high doses of the latter medication.

According to the latest 2015 ESC Guidelines for the diagnosis and management of pericardial diseases, a stepwise approach, including four consecutive steps, has been proposed for the treatment of recurrent pericarditis (Figure 2) [1]. The first step includes exercise restriction, aspirin/non-steroidal anti-inflammatory drugs (NSAIDs—ibuprofen, naproxen, and indomethacin), plus colchicine and gastroprotection. At this point, it should be emphasized that, based on high-quality clinical data, colchicine can halve the recurrence rate either after the first or subsequent episodes of pericarditis [64,65,66,67,68]. In case of failure of the first step treatment strategy, the administration of glucocorticoids along with colchicine is recommended as the second step. In contrast with the previous concepts, high doses of steroids should be avoided since a higher rate of side effects accompanies them without any additional clinical benefit regarding recurrence rate [69]. Thus, the daily dose of steroids should be, according to the current concepts, between 0.2 and 0.5 mg/kg of prednisone (or an equivalent dose of an alternative steroid). After normalizing CRP levels, the full dose should be gradually reduced, with tapering being very slow (several weeks) for doses less than 5 mg [1,36,70]. The great majority of patients with chronic disease and multiple recurrences receive this second-step regimen and, most of the time, depict steroid dependency with an inability to discontinue glucocorticoids without developing a recurrence [1,27]. According to the Guidelines, an additional intermediate step between the second and third consists of administering triple therapy (aspirin/NSAIDs, glucocorticoids, and colchicine) and adopting moderate doses of each class in an effort to enhance efficacy without a substantial risk of side effects [1,71]. If the above-mentioned regimen does not work, then the third step includes the addition of an immunosuppressant/immunomodulatory agent such as intravenous human immunoglobulins (IVIG), azathioprine, or anakinra, which, at the time that the 2015 Guidelines were published, was the only IL-1 blocker available in clinical practice [1]. However, the clinical data on the efficacy of the latter agents were scant and derived from case reports, case series, and retrospective studies [1,30,32]. Moreover, in the case of azathioprine, most of the patients had not received colchicine before azathioprine commencement, according to contemporary concepts [30]. At present, immunosuppressive treatments (excluding IL-1 blockers, which will be individually addressed subsequently) may have a role in recurrent pericarditis patients presenting with moderately elevated or near normal CRP levels (namely with the so-called autoimmune phenotype) as steroid-sparing agents [71]. Another steroid-sparing agent that has been tested with promising results in patients with glucocorticoid-dependent colchicine-resistant idiopathic recurrent pericarditis and elevated CRP is hydroxychloroquine [25]. Finally, in patients who are not able to tolerate anti-inflammatory treatment due to troublesome side effects or who do not wish to receive long-standing treatments, pericardiectomy is a valid alternative and can be safely performed with good results in centers with expertise in this type of intervention [1,72].

## 4. Mechanism of Action of Available IL-1 Blockers

At present, the currently available and tested in clinical practice anti-IL-1α and 1β blockers encompass anakinra, rilonacept, and goflikicept [73,74,75,76]. Canakinumab is included in the broader IL-1 family blocks selectively IL-1β [77]. The experience with the latter medication in recurrent pericarditis is very limited and consists of occasional case reports and small case series where canakinumab overall depicted a controversial efficacy (in most instances failed to achieve stable control of pericarditis) at least in idiopathic forms of pericarditis [78,79,80,81]. Inhibition of both IL-1α and 1β seems crucial to abate inflammation in acute pericarditis [81]. For the above reasons, canakinumab will not be further addressed as a potential option for recurrent pericarditis in the present review. Concerning the rest of the IL-1 blockers, anakinra is a recombinant human IL-1 receptor antagonist (IL-1Ra) that binds to the IL-1 receptor, competing with and inhibiting the activity of IL-1 alpha and beta. Inflammatory stimuli induce IL-1 production and mediate several physiological mechanisms, such as inflammation and immunological reactions. By antagonizing the IL-1 receptor, anakinra blocks the effects of IL-1a and IL-1β and accordingly prevents the cascade of sterile inflammation in a pathological state and in the assembly of the inflammasome [32,81].

Rilonacept, on the other hand, blocks IL-1 signaling by acting as a soluble decoy receptor that binds both IL-1α and IL-1β, thus preventing their interplay with cell surface receptors, disrupting the cycle of autoinflammation encountered in recurrent pericarditis, at least in some cases [75].

Finally, goflikicept is a heterodimeric fusion protein capable of a high-affinity binding to human IL-1α and IL-1β and affecting their signaling pathways as depicted in relevant preclinical studies [76]

## 5. Contemporary Clinical Experience with IL 1 Blockers in Recurrent Pericarditis

The first report of IL-1 blockers for the treatment of recurrent pericarditis dates back to 2009, when an Italian group administered anakinra in three children with refractory idiopathic recurrent pericarditis [48]. It should be emphasized that chronic steroid administration in pediatric patients is very problematic due to the serious side effects in this patient population, with growth impairment being the major concern. Unfortunately, symptoms recurred after anakinra discontinuation and abated once again after re-administration of the drug without further recurrences while on treatment. This case series should be viewed as a proof-of-concept study that suggested that idiopathic recurrent pericarditis (or at least some forms of the disease) may be included among autoinflammatory diseases. Moreover, it has been shown for the first time that IL-1 inhibition with anakinra provides a favorable cost–benefit ratio.

Three years later, in 2012, a Greek research group published its preliminary experience on the use of anakinra in adults, this time patients with refractory recurrent pericarditis [82]. Similar to the pediatric patients, anakinra depicted a similar efficacy and safety in adults. Moreover, the need for long treatment duration was put forward in this series of three patients. In a case series published shortly afterward, anakinra depicted excellent efficacy without remarkable side effects. Specific symptoms usually resolve within 48 h, CRP normalizes within 1 week, and glucocorticoids are discontinued within 4–6 weeks [42].

Taking into account the very encouraging initial experience with the use of anakinra in recurrent pericarditis presenting with an anti-inflammatory phenotype, a few years later, the first randomized placebo-controlled trial in this context was published [73]. Specifically, in the AIRTRIP trial, which was published in 2016, 21 patients with colchicine resistance and corticosteroid dependence were included. In this double-blind, placebo-controlled, randomized withdrawal trial, 10 patients were assigned to a placebo and 11 to anakinra. The primary outcomes were recurrent pericarditis and time to recurrence after randomization, and all patients were followed up for 12 months. Despite the low number of patients enrolled, a statistically significant benefit of anakinra was observed. Specifically, recurrent pericarditis occurred in 9 out of 10 patients assigned to the placebo (90%; incidence rate, 2.06% of patients per year) and in 2 out of 11 patients (18.2%; incidence rate, 0.11% of patients per year) assigned to anakinra (incidence rate difference of −1.95% (95% CI, −3.3% to −0.6%). The median flare-free survival (time to flare) was 72 (interquartile range, 64–150) days after randomization in the placebo group and was not reached in the anakinra group (*p* < 0.001). The most common side effects included local skin reactions and transaminasemia; however, no patient permanently discontinued the active drug. According to this study protocol, anakinra was administered at 2 mg/kg per day (up to 100 mg) for 2 months, and then 11 patients were randomized to continue anakinra while the remainder (11 patients) switched to the placebo (*n* = 10) for 6 additional months or until a pericarditis recurrence [73].

The encouraging results observed in the AIRTRIP trial were subsequently confirmed in the real world, as shown in the IRAP (International Registry of Anakinra for Pericarditis) registry [74,83]. The largest real-world registry published to date is IRAP (International Registry of Anakinra for Pericarditis) [74]. The registry in question included 224 consecutive patients with glucocorticoid-dependent colchicine-resistant recurrent pericarditis treated with anakinra. The primary outcome of this work consisted of the pericarditis recurrence rate after treatment, whereas secondary endpoints included emergency department visits, hospitalizations, corticosteroid use, and adverse events. The mean age of the study population was 46 ± 14 years; 63% were females, and the etiology of pericarditis was idiopathic in 75% of cases. The median duration of the disease was 17 months (interquartile range 9–33), while CRP elevation was recorded in 91% of patients and pericardial effusion in 88%. According to the results of this investigation, a sixfold (2.33–0.39 per patient per year) reduction in pericarditis recurrences was detected after a median treatment of 6 months. Regarding the secondary endpoints, hospitalizations were reduced 7-fold (0.99–0.13 per patient per year), and emergency department admissions were reduced 11-fold (1.08–0.10 per patient per year). Remarkably, glucocorticoid use decreased from 80% to 27%, *p* < 0.001. The most common side effects were transient skin reactions in 38% of cases, with permanent drug discontinuation for side effects being recorded in a minority of cases (~3%). The lowest risk of recurrences was detected in patients receiving a full dose regimen for at least 3 months, followed by a tapering period of over 3 months. Thus, in the real-world setting in patients with recurrent pericarditis, anakinra has been proven an effective, safe treatment in terms of reducing recurrences, emergency department admissions, and hospitalizations in patients with glucocorticoid-dependent colchicine-resistant recurrent pericarditis [74].

In 2021, the results on the efficacy and safety of rilonacept, an IL-1α and IL-1β cytokine trap in patients with recurrent pericarditis (≥2 recurrences), were published [75]. Rilonacept has been tested in a phase 3 event-driven multicenter randomized withdrawal trial. Rilonacept was given subcutaneously at a loading dose of 320 mg and 160 mg weekly. The prerequisite for enrollment was the presence of an acute event in a patient with at least two recurrences of pericarditis along with CRP elevation. The primary endpoint of the investigation was the time to the first pericarditis recurrence, and safety issues were also addressed. The advantage of rilonacept relies on the once-weekly administration required compared to the daily injections recommended for anakinra, which is important in compliance and quality of life, provided that the two medications are comparable in terms of efficacy and safety. The number of patients randomized after the initial run-in period was 61. The median time to the first adjudicated recurrence in the placebo group was 8.6 weeks, whereas the recurrent events were too few to calculate the median time to the first recurrence. The median duration of exposure to rilonacept was 9 months. Compared to the placebo, Rilonacept was related to a significantly lower risk of recurrent pericarditis, with the hazard ratio being calculated to 0.04 (*p* < 0.001). In particular, 2 out of 30 patients assigned to the rilonacept group (7%) and 23 out of 31 patients (74%) in the placebo group developed a recurrence during follow-up. The drug was proven to be safe in terms of safety, with the most common adverse effects being local skin irritation, as with anakinra and upper respiratory tract infections. Drug discontinuation was accomplished in 4 out of 81 screen patients during the run-in period. Based on the results of this study, rilonacept was the first IL-1 1 blocker that received FDA approval to reduce the risk of recurrences in adults and children older than 12 years with recurrent pericarditis [75].

Very recently, an additional IL-1 blocker, namely goflikicept, was tested in patients with recurrent pericarditis [76]. The advantage of this medication consisted of the longer interval between doses compared to the previous medications, which was 15 days. The drug has been administered in the setting of a phase II/III two-center open-label clinical study with or without recurrence at the time of enrollment. As in previous investigations, the design consisted of a randomized, placebo-controlled, withdrawn approach. The endpoint of the study was time to first pericarditis recurrence. The study population consisted of 20 patients, with 10 patients in each group. Goflikicept was administered at a dose of 160 mg at week 0, then 80 mg at weeks 1 and 2, and thereafter 80 mg every 2 weeks. Pericarditis recurrence was observed in 9 out of 10 patients in the placebo group but in no patient in the active medication group 24 weeks after randomization. Notably, no deaths or new safety issues were observed in the goflikicept group, indicating a favorable risk–benefit ratio [76].

In randomized controlled trials, the clinical benefits of IL-1 blockers in patients with recurrent pericarditis have been shown predominantly in cases of “idiopathic recurrent pericarditis” and post-cardiac injury syndrome [73,75,76]. However, in real-world acute pericarditis, patients with alternative specific etiologies such as autoimmune diseases, autoinflammatory diseases, and occasionally radiation and traumatic pericarditis have been additionally included [74]. In recent years, an emerging indication of IL-1 blockage is the reversal of constrictive/effusive constrictive pericarditis in patients with incessant pericarditis. The latter subset of patients has been shown to progress to constriction in 28% of cases over time [21]. Administration of anakinra in patients with constrictive pericarditis following incessant pericarditis resulted in the complete resolution of pericardial constriction in 63% of cases within a median of 1.2 months [21].

The main characteristics, indications, and side effects of these agents, as well as the dosing schemes in the setting of pericarditis, are presented in Table 1.

## 6. Adverse Effects Related to IL-1 Blockers

IL-1 blockers, taking into consideration the literature data from their administration in pericarditis patients as well as in other clinical settings, are generally safe drugs with very few contraindications, such as hypersensitivity to the drug and marked neutropenia.

The accumulated experience with their use in clinical practice dates more than 20 years, with anakinra being the most widely used medication for the longest period of time, while relevant data on goflikicept are scant [76,84]. The most common side effects related to the latter medications consist of skin reactions (erythema) and occasionally pain at the injection site. Local reactions are most commonly observed with anakinra (38–95%) and rilonacept (34–60%) and less frequently with goflikicept (~20%) [32,73,74,76,81]. Skin reactions usually appear within 1–2 weeks for the first administration and spontaneously abate within a few weeks [81] with the help of a few helpful treatments: warming the syringes to room temperature (they are stored in the refrigerator), alternating injection sites, local ice a few minutes after the injection, and local (or systemic in most serious reactions) antihistamines and corticosteroids [32,81].

Other side effects include neutropenia reported in 1–9% of cases depending on the individual agent, transaminasemia (up to 14% with anakinra and 4% with rilonacept), arthralgias/myalgias (up to 8% with anakinra and 12% with rilonacept) [32,73,74,81]. The reported rate of blood lipids elevation (generally mild) is up to 8% with rilonacept and up to 18% with goflikicept (hypercholesterolemia), and is also possible with anakinra [76,81].

Infections, mostly upper respiratory or skin infections, are observed in approximately 3% of cases after administration of anakinra [81]. The rate of infections and infestations reported in the goflikicept trial was ~22% [76]. Further details on IL-1-related infections will be provided in the following section.

In general, severe side effects are reported in 1–4% of cases in patients receiving anakinra and up to 6% of patients receiving rilonacept [81]. Permanent discontinuation due to adverse events was required in 3% of cases overall [81]. Data on goflikicept are limited [76]. Nevertheless, as already mentioned, no deaths or new safety signals were reported in patients with recurrent pericarditis receiving goflikicept [76].

## 7. Treatment Protocols of IL-1 Blockers

There is no universally accepted treatment protocol concerning interleukin-1 blocker administration in refractory recurrent pericarditis. In particular, although the dose of each IL-1 blocker has been established, experts differ between the full dose regimen duration and the tapering protocol. Most of the discussion is related to anakinra, whereas data on rilonacept are limited, and those on goflikicept are very scant.

In the specific context of anakinra, according to the IRAP registry findings, the full-dose regimen period of administration of over 3 months followed by a tapering period of at least 3 months were the treatment strategies associated with a lower risk of recurrence [74]. It should be emphasized that treatment protocols with anakinra depend mainly on the local institutional expertise and that no head-to-head comparison between different protocols in terms of efficacy, safety, and recurrence rate has been performed so far. In the real world, IRAP showed that the median duration of anakinra treatment is 6 months (IQR 3–12), while the median duration of the tapering period is 3 months (IQR 0–6) [74].

The impact of dose tapering on the recurrence rate has been shown early after the administration of anakinra in recurrent pericarditis patients. In particular, abrupt discontinuation of the medication after a period of full-dose administration was accompanied by recurrences in up to 70% of cases shortly (in general within 1 month) [42]. With gradual tapering, the rate of recurrences has been lowered up to 50%, with most of the recurrences being observed when the number of injections per month is reduced below 12 (<3 weekly) [26,28,42,74,85].

According to our institutional protocols, an anakinra full-dose regimen (one subcutaneous injection daily) is administered for 6 months, and then one injection per week is omitted every month. Recently, a group of experts proposed a full dose of anakinra (100 mg/d) for at least 2 months, and in case of stable remission, injections are administered on alternative days. In the absence of pericarditis flair in the next 2 months the dose is reduced further to every third day, and so forth in a 2-month time period fashion [86]. Unfortunately, despite the accumulating experience with anakinra treatment protocols, the rate of patients who are able to discontinue anakinra is disappointing. In a recent study conducted on a pediatric population, only ~15% of patients were able to discontinue the IL-1 blockade in a follow-up period of 2.6 years [87]. 

The data on the rilonacept treatment protocol are less robust compared to anakinra, and almost all results from the RHAPSODY trial are lacking, while real-world data are still lacking [75]. Based on the current experience and the pharmacokinetics of rilonacept, the minimal duration of the treatment is 6–8 months [81]. Based on experts’ opinion, however, treatment should be prolonged at least for 1 year, usually up to 18 months [75,81]. Given the long half-life time of the molecule (~7 days), no dose tapering is recommended after the full dose regimen, and cardiac MRI, which may detect residual pericardial inflammation, may guide treatment duration [86]. Cardiac MRI has the ability to characterize tissue, allowing objective identification of ongoing pericardial inflammation based on the detection of pericardial edema and late gadolinium enhancement. It should be performed every 6–12 months, when possible, to assist tapering and/or discontinuation of anti-IL-1 agents in cases where the remission state is not well established, as may occur in symptomatic patients despite normal or near-normal CRP [86]. 

Recently, the long-term extension results of RHAPSODY provided further insights into efficacy, safety, and clinical decision making. Remarkably, treatment suspension after 18 months of treatment discontinuation caused pericarditis recurrence in six out of eight patients (75%) [88]. 

Putting the data gathered from anakinra and rilonacept treatment in glucocorticoid-dependent colchicine-resistant pericarditis together, it is clear that both IL-1 blockers are extremely efficacious in achieving pericarditis inflammation while on treatment with a very good safety profile. Unfortunately, they do not seem to cure recurrent pericarditis since, in the great majority of patients, recurrences appear during dose tapering or drug discontinuation [26,28,85,87]. Thus, despite the big step forward, the cure for recurrent pericarditis still constitutes an unmet need. 

Finally, data on goflikicept are insufficient and are offered from an open-label randomized trial that enrolled 20 patients (10 on goflikicept) who received treatment for 24 weeks and then followed up in terms of safety for 8 additional weeks [76]. Thus, no recommendations can be provided at present for goflikicept. 

## 8. Use of IL-1 Blockers in Specific Clinical Scenarios 

### 8.1. Conception, Pregnancy, and Lactation 

Women presenting with an autoinflammatory disease are at increased risk of pregnancy complications owing to the systemic effects of inflammation. The safety of the perinatal use of IL-1 inhibitors in such patients is still under debate. The available literature data appear encouraging even though the supporting studies are few and mostly retrospective. 

The most recent international guidelines mainly deal with the use of anakinra and canakinumab, as there are only scarce data on rilonacept [89,90]. Because these medications contain the IgG1 component, their placental transfer especially in the first trimester, should be considered negligible. Published data on maternal exposure to anakinra and canakinumab showed very few congenital anomalies (chiefly renal agenesis) and oligohydramnios, even though the latter may also be associated with maternal hyperthermia [91,92]. A recent systematic review on the safety of blocking the IL-1 pathway in pregnancy assessed 88 different pregnancies and did not associate the perinatal use of anakinra or canakinumab with an increased risk for complications, such as increased rate of miscarriage, preterm deliveries, or congenital disabilities [93]. Taken together, despite the insufficient evidence for someone to be confident, the data appear reassuring for the use of IL-1 inhibitors during conception and pregnancy, especially during the first trimester. Careful consideration of pregnancy-compatible alternative therapies and balancing the likelihood of severe maternal disease relapse should inform a shared decision about their use during the second and third trimesters. 

Up to this date, there are no data on IL-1 inhibitor transfer into breast milk, and as these drugs have a high molecular weight, it is unlikely that clinically significant amounts can be transferred. Furthermore, there is an even lower risk of any substantial quantity passing through the infants’ gastrointestinal tract to their bloodstream. Studies of breastmilk exposure to anakinra and canakinumab have not shown significant adverse effects that could be attributed to the drug, including serious infections and developmental defects [91,94]. The recent British Society of Rheumatology (BSR) guidelines have deemed maternal exposure to IL-1 blockers compatible with lactation [90]. If a woman needs to be treated with anakinra, there is no reason for her to stop breastfeeding. 

Parental exposure to anakinra or canakinumab does not seem to affect the outcome of the resulting pregnancies. Youngstein et al. have reported five such cases for each medication without any adverse effects [91]. Both the 2020 American College of Rheumatology (ACR) and the 2023 BSR Reproductive Guidelines [89,90] conditionally recommend continuing anakinra in men who are planning to have a child [89,90]. 

### 8.2. Malignancy

The potential effects of blocking the IL-1 pathway on the vulnerability to new or recurrent malignancies have been a concern when treating patients with autoinflammatory conditions. 

In the setting of anakinra use in RA, where there is longer-term data available from extension trials, the overall incidence of malignancies (1.2 events/100 patient-years) was lower than the expected rates for the general population [95]. Further support for the safety of anakinra was recently provided by a meta-analysis of studies of biologic DMARDs in rheumatoid arthritis and history of prior malignancy [96]. The absolute incidence rate of developing new or recurrent cancer was 32.3 per 1000 patient-years in the anakinra-exposed group, similar to conventional and other biological DMARDs. 

A rather interesting notion in the recent literature is that blocking the IL-1 pathway may be a therapeutic option in malignancy, e.g., colorectal and breast cancer [97,98]. In an analysis of the CANTOS trial, incident new lung cancer diagnosis, lung, and total cancer mortality were significantly lower in the canakinumab-treated population compared to placebo, suggesting that the anti-inflammatory effect of blocking IL-1β innate immunity may protect from the development of lung cancer [99]. 

When combined, these data provide reassurance for the safety of IL-1 inhibitors when considering malignancy risk. 

### 8.3. Infection 

Several randomized controlled trials have assessed the safety of long-term treatment of IL-1 inhibitors with regard to infection across various indications. It has to be noted that there is a difference in the attributed risk depending on the indication these drugs are used for. For example, the cumulative risk for serious infections in patients with rheumatoid arthritis treated with anakinra for three years has been reported to be 5.4/100 patient-years (2.87/100 patient-years in patients not receiving corticosteroids at baseline). In contrast, the same risk is much lower in patients suffering from other autoinflammatory syndromes and closer to the risk of the general population [95,100]. In the published literature, there are only a few case reports of opportunistic infections, reactivation of tuberculosis, or varicella-zoster virus infections in anakinra-treated patients. Furthermore, in the CANTOS trial, where patients with previous acute myocardial infarction and elevated CRP were treated with canakinumab or a placebo, the risk for fatal infection was generally low but higher in comparison to the placebo (0.31 vs. 0.18 per 100 patient-years, respectively). Conversely, the rate of opportunistic infections was not different in the compared groups [77].

Anakinra has been reported to be associated with a low rate of serum aminotransferase elevations and rarely with acute liver injury, both of which have been shown to resolve following its discontinuation [101]. However, when considering anakinra treatment in the setting of viral hepatitis, data are particularly scarce. The risk of hepatitis B virus reactivation (HBVr) with the use of IL-1 inhibition is difficult to estimate, as only very few cases of HBVr have been reported in patients with resolved HBV infection [102]. The current recommendation is to follow the practices applied when using other biological DMARDs, i.e., antiviral prophylaxis in chronic HBV infection and careful monitoring of aminotransferases and HBV viral load in resolved HBV infection [103]. As there is no risk for reactivation of chronic hepatitis C infection and the risk of anakinra hepatoxicity is low, there is no contraindication for its use in these patients, provided that the liver function is not significantly impaired. 

Overall, the use of IL-1 inhibitors may be associated with a moderate increase in the overall risk of infection, but these infections are generally of mild to moderate severity [104]. Of particular note and compared with other disease-modifying anti-rheumatic drugs (DMARDs), and certainly with corticosteroids, anakinra has rarely been associated with serious infections (such as hospitalized pneumonia or pyelonephritis). In terms of safety, an added benefit of anakinra is its short half-life, making it a preferable choice in patients with increased infection risk due to age, indication, or comorbidities. Finally, opportunistic infections, tuberculosis, and herpes zoster are rarely reported in patients treated with IL-1 inhibitors. 

Anakinra was one of the first medications studied off-label for its efficacy in treating COVID-19 and the associated “cytokine storm”. Following the results of the SAVE-MORE trial, anakinra was approved for the treatment of COVID-19 in adult patients with pneumonia requiring supplemental oxygen and who are at risk of developing severe respiratory failure, as determined by elevated suPAR (soluble urokinase plasminogen activator receptor) at values > 6 ng/mL, providing a potential benefit in the early stages of SARS-CoV-2 infection [105]. However, in more recent systematic reviews and meta-analyses, anakinra and canakinumab exhibited little to no effect on mortality, safety outcomes, and disease progression in adult hospitalized patients with SARS-CoV-2 infection [106,107]. Given that patients with autoinflammatory conditions are at increased risk for relapse after discontinuation of IL-1 inhibitors, the authors would recommend conditionally continuing anakinra in patients contracting COVID-19 while on long-term treatment. 

## 9. Clinical Implications—Future Perspectives 

Until approximately 10 years ago, available treatments for refractory recurrent pericarditis were very limited. Most importantly, patients with long-standing disease and colchicine resistance were subjected to sometimes devastating side effects mainly due to glucocorticoid dependency. 

The introduction of IL-1 blockers in treating these unfortunate patients was a fundamental change in managing this condition with a clear positive cost–benefit ratio in terms of glucocorticoid discontinuation, quality of life, efficacy, and safety profile. Nevertheless, despite the indisputable steps forward, additional issues should be better defined in the near future, the most important being the adoption of universally accepted protocols in terms of treatment duration and tapering process. 

The development and introduction in clinical practice of additional IL-1 agents with even longer time intervals between injections is eagerly awaited. Last but not least, the final goal is the complete decodification of the pathogenetic mechanisms of recurrent pericarditis and the advent of clinical practice tailored to the patient’s treatments to permanently abate the disease. 

Concerns related to the widespread use of IL-1 blockers in the real world are mainly related to their high cost, which holds especially true for rilonacept, the off-label use of anakinra in idiopathic recurrent pericarditis and their unavailability in several countries. On the other hand, their high efficacy and good safety profile, as well as the overall cost-effectiveness analysis, should be taken into account in clinical decision making. 

Finally, the best benefit/risk profile of the commercially available IL-1 blockers in children and adults with recurrent pericarditis remains to be addressed, taking into account the absence of a head-to-head comparison among the available drugs. 

## 10. Conclusions 

IL-1 blockers are a paradigm shift in the treatment of patients with glucocorticoid-dependent colchicine-resistant recurrent pericarditis, a disease where available treatment options are scant. Based on the available evidence, IL-1 blockers are mostly reserved for patients with the auto-inflammatory pericarditis phenotype (namely with striking CRP elevation, fever, and serositis), depicting recurrences despite an optimal treatment of pericarditis with first- or second-line agents. Possible candidates are patients unable to reduce the prednisone dose to <5–7.5 mg/day and those with or at risk for glucocorticoid-related adverse effects [28]. IL-1 blockers have a very good risk–benefit profile, but since they do not cure the disease, the possibility of prolonged treatment with those agents (occasionally >10 years for anakinra in our institutional experience) should be discussed with the patient. Nevertheless, despite these limitations related to the use of IL-1 blockers, they are still the most valid and probably unique treatment option in difficult patients with devastating side effects due to steroid dependence. According to real-world experience, they allow sustainable remissions (at least with the full dose regimen), prompt discontinuation of steroid treatment in less than 6 weeks in most instances, and a remarkable improvement in patient-reported health-related quality of life, global symptom severity, sleep, and pain, while on treatment [74,108]. Last but not least, the full-dose treatment period and weaning strategies for each agent should be further investigated. 

## Figures and Tables

**Table 1:** Main characteristics and dosing considerations for IL-1-targeted therapeutics in pericarditis.

	Anakinra	Rilonacept	Goflikicept
Mechanism of action	Recombinant human IL-1Ra	IL-1α and IL-1β trap	Heterodimeric anti-IL-1α and anti-IL-1β inhibitor
Half-life	4–6 h	7 days	10 days
Route of administration	SC or IV	SC	SC
Route of elimination	Mostly renal	Reticuloendothelial system	Not available
Main indications	CAPS, FMF, Still’s disease, recurrent pericarditis, rheumatoid arthritis (in scarce clinical scenarios)	CAPS, recurrent pericarditis	Recurrent pericarditis-
Most common side effects	Injection site reactions, hepatitis, infections	Injection site reactions, neutropenia, infections, dyslipidemia	Injection site reactions, neutropenia, infections, dyslipidemia
Pericarditis treatment regimen			
Dose	1–2 mg/kg/day up to 100 mg/day	Loading: 320 mg on first day (or 4.4 mg/kg if <18 years of age) Maintenance: 160 mg weekly (or 2.2 mg/kg if <18 years of age)	Loading: 160 mg (Week 0) Maintenance: 80 mg (Week 1)—80 mg (Week 2)—80 mg every two weeks
Duration	Usually 3–6 months	At least 6–8 months	Limited data
Tapering	At least 3–6 months	No data/Probably not required	No data/Probably not required

IL-1Ra: interleukin-1 receptor antagonist; SC: subcutaneous; IV: intravenous; CAPS: cryopyrin-associated autoinflammatory syndrome; and FMF: familial Mediterranean fever.

## Data Availability

Not applicable.
